# Moral Dilemmas and Existential Issues Encountered Both in Psychotherapy and Philosophical Counseling Practices

**DOI:** 10.5964/ejop.v11i3.1010

**Published:** 2015-08-20

**Authors:** Beatrice A. Popescu

**Affiliations:** a Faculty of Philosophy, University of Bucharest, Bucharest, Romania; Aalborg University, Aalborg, Denmark

**Keywords:** cognitive behavioral therapy, philosophical counseling, moral dilemmas, existential issues, meaning of life

## Abstract

This paper stems from clinical observations and empirical data collected in the therapy room over six years. It investigates the relationship between psychotherapy and philosophical counseling, proposing an integrative model of counseling. During cognitive behavior therapy sessions with clients who turn to therapy in order to solve their clinical issues, the author noticed that behind most of the invalidating symptoms classified by the DSM-5 as depression, anxiety, hypochondriac and phobic complaints, usually lies a lack of existential meaning or existential scope and clients are also tormented by moral dilemmas. Following the anamnestic interview and the psychological evaluation, rarely the depression or anxiety diagnosed on Axis I is purely just a sum of invalidating symptoms, which may disappear if treated symptomatically. When applying the Sentence Completion Test, an 80 items test of psychodynamic origin and high-face validity, most of the clients report an entire plethora of conscious or unconscious motivations, distorted cognitions or irrational thinking but also grave existential themes such as scope or meaning of life, professional identity, fear of death, solitude and loneliness, freedom of choice and liberty. Same issues are approached in the philosophical counseling practice, but no systematic research has been done yet in the field. Future research and investigation is needed in order to assess the importance of moral dilemmas and existential issues in both practices.

## Introduction

The main topics of this paper refer to the moral dilemmas and existential issues encountered both in psychotherapy and philosophical counseling. Moral dilemmas may vary from personal dilemmas linked to moral relativism, dilemmas related to academic performance, drugs and alcohol, up to a wide range of business ethical dilemmas. Existential issues cover topics such as: scope or meaning of life, professional identity, fear of death, solitude and loneliness, freedom of choice and liberty. Existentialist themes may be approached as well in existential psychotherapy and in philosophical counseling sessions, following different traditions or school of thoughts. Psychotherapy practice in general gives rise to a lot of ethical and moral dilemmas and also deals with a large sum of existential issues. The same happens in the philosophical counseling practice. In this paper, I argue that both the mental health dimension and the moral dimension can be brought into the same integrative model of counseling, starting from the limitations of both fields. The paper also aims to theorize some of the main ethical dilemmas encountered both in psychotherapy and philosophical counseling practices, especially the dilemmas raised by the clients or counselees, which sometimes can be noticed in the first session of the evaluation. The selection process of clients or counselees is also discussed, since it is crucial for the success of both therapeutic and counseling approaches.

## Psychotherapy and Philosophical Counseling or the Need for an Integrative Model of Counseling

Psychotherapy and philosophical counseling as distinct domains have been developing in parallel for a few decades. Even though philosophical counseling is much newer than psychological counseling or psychotherapy in the helping professions field, philosophical counselors, especially those who have mental health training such as Tim LeBon, Eliott D. Cohen, Mike W. Martin, John Mills and are also trained in psychology except from philosophy, value also the methods of psychotherapy, mainly those used in cognitive behavior psychotherapy, rational emotive behavior therapy and existential psychotherapy. The methodological impact of psychoanalysis on philosophical counseling is rather limited. However, existential psychotherapy as a theoretical frame reminds us that philosophy, in this case existentialism does not only mean any kind of reflection, discussion or lecturing, but it is also a way of life and an existential attitude. (Hadot, 1995).

In Schlomit Schuster’s paper, “Philosophical Counseling and Rationality”, she also mentions two articles in the International Journal of Applied Philosophy that relate philosophical counseling to Albert Ellis's Rational Emotive Behavior Therapy (REBT). Another author, the philosopher Roger Paden, argues that PC should be made even more similar to REBT. (Schuster, 1999). In Paden's paper, “Defining Philosophical Counseling”, philosophical counseling is considered therapy, yet he considers PC different “from psychotherapy and pastoral counseling, very similar to REBT but lacking a fixed paradigm” (Paden, 1998, p. 10).

Not only the theoretical proximity of the two domains, with psychology initially evolving from philosophy, makes us consider their synergistic approach, but also the methods and techniques used in both types of practices (psychotherapy and philosophical counseling) are very similar, with a focus on the methods used in cognitive behavior psychotherapy (CBT) and rational emotive behavior therapy (REBT). Cognitive techniques commonly used in both practices are: rational analysis (Anderson, 1991), disputing irrational beliefs in Windy Dryden’s ABCDE scheme (Ellis & Dryden, 2007), changing one’s language, challenging client’s or counselee’s worldview (Lahav, 1995), Nelson’s method of Socratic dialogue (Kleinknecht & Neißer, 1994), identifying the cognitive distortions (Burns, 1989), common irrational beliefs (Ellis, 1991) or logical errors, cognitive reframing, formulating practical syllogisms, as in Cohen’s Logic-Based Therapy, mindfulness meditation (drawn from the Buddhist tradition) and critical thinking (Feltham, 2010). Other techniques discovered by De Haas based on phenomenological tradition are: phenomenological *epoché* and reduction, by asking open and disenchanting questions and also language game analysis. (De Haas, 2011).

Even more, the ABC schema created by Albert Ellis, the father of rational emotive behavior therapy, one of the few evidence-based or bona fides psychotherapies used to treat a wide range of problems, is heavily relying on Stoics’ thinking. It is inspired by Epictetus’s *Enchiridion*: “Men are disturbed not by things, but by the view which they take of them.” Ellis describes people’s interpretations of external events as being influenced by core irrational beliefs (Ellis, 1991). I can argue in this paper that an eclectic model of counseling can exist, based on the fact that “cognitive-behavioral approaches, Transactional Analysis, cognitive behavior therapy (CBT) and rational emotive behavior therapy (REBT) among others, brought forth a mixed bag of philosophical assumptions” (Cohen, 2013, p. 11).

Looking at the analytical and methodical jargon of most philosophical counselors, Leon De Haas notices that it is not entirely derived from psychology, but also from philosophy, existentialism, stoicism or non-Western sources of ‘sage wisdom’ such as Buddhism (De Haas, 2011). Tim LeBon, an UK cognitive behavior psychotherapist and philosopher, believes certain therapies, even evidence-based ones (such as cognitive behavioral therapy), don't go far enough in helping their clients.

For instance, if you are anxious about your relationship, a cognitive therapist would try to dispute your catastrophizing and jump to conclusions to make you feel less anxious. A philosophical counselor would do this, but would also look for existential meaning in your anxiety - perhaps you really don't want to be in the relationship and that is what your anxiety is telling you (LeBon, 2001, p. 134).

However, not all the philosophical counselors are seduced by this “marriage” of philosophical counseling with psychotherapy. Lydia Amir tries to delimit herself from this view, stating that

“most people come to philosophical counseling in order to solve some predicament, usually after also having undergone psychological counseling” (Amir, 2004a, p. 2), urging philosophical counseling practitioners to claim “complete independence from psychology” (Amir, 2004a, p. 7).

Despite the inherent controversies the emergence of the new philosophical counseling field has created, especially when it comes to training requirements of its practitioners, a lot of voices still regard the “be wedding of philosophical and psychological practices” (Cohen, 2004, p. 1) as a beneficial one, especially in the area of improving critical thinking of the psychological practitioners, far too long embedded in their concepts of emotionality and attachment theories and relying too little on rationality and logic. Peter Raabe believes that “the attainment of personal autonomy – thinking for oneself and being responsible for oneself – are particularly strong themes in both philosophy and most forms of psychotherapies” (Raabe, 2013, p. 162). However, Raabe’s opinion on mental illnesses (as mind diseases, not brain diseases) and medicalization is developed based on the theories and assumptions of two famous psychiatrists: Thomas Szasz and Peter Breggin. While Breggin advocates for an empathic therapy as opposed of using medication, Raabe militates for prescribing the client philosophical therapy instead of medication. Regardless of the efficacy of medication for a variety of conditions considered today as treatable only in the medical model paradigm (schizophrenia and other psychoses), all practitioners and the vast majority of psychiatrists consider psychotherapy necessary at least as a support for compliance to medical treatment. However, latest meta-analyses (Fournier et al., 2010) found that for patients with mild or moderate depression symptoms, drug response (compared with placebo), may be minimal or nonexistent, which leaves us with the supposition that there are a lot of ”clinical” situations in which medication is not considered the first choice.

Recent research in the area of the evidence-based psychotherapies (cognitive behavior psychotherapy and rational emotive behavior therapy) are also supporting the view that, with very few exceptions of childhood trauma, no matter what happened in the client’s past, focusing on present (mindfulness training) and future (solution focused training) are valuable steps for the success of the process and for the improvement of the person’s state of mind.

## Selection of Clients, a Conundrum for Both Practices

This paper investigates the attitudes of philosophical counselors and psychotherapists in private practice towards various factors concerning the selection of clients. Psychotherapists and counselors are influenced not only by diagnostic criteria, but also by other factors relating to the client. The most important selection criteria are: desire for change, motivation for therapy or counseling and evidence of psychopathology, although there is no clear consensus about the criteria overall.

In both practices, philosophical counseling and psychotherapy, the selection of clients may be difficult, but it is a crucial task for the success of these processes. Looking at the psychotherapy field, when the psychotherapist is using validated psychological assessment tools for detecting the client’s problems during the anamnestic interview, the practitioner may happen to diagnose as well a psychiatric disorder, such as paranoid schizophrenia or delusional disorder. However, the non-maleficence principle of bioethics stated in the “*Ethical Framework for Good Practice in Counselling and Psychotherapy*” (British Association for Counselling & Psychotherapy, 2012) urges the psychotherapist to refer this particular client to a psychiatrist and, only then, the client can also enter in a psychotherapeutic process with the best chances of recovery. Therefore, there will always be an overlapping of interventions in the case of a psychiatric patient: a psychiatrist’s intervention and a psychotherapist’s intervention, preferably one trained in an evidence-based psychotherapy (as CBT) and practicing a bona-fide therapy.

Looking at the philosophical counseling field, as Lou Marinoff acknowledged in one of his initial works, “Therapy for the sane”, that philosophical counseling should address normal people who don’t have psychiatric problems that need to be dealt with by using medication (Marinoff, 1999, 2003). At first glance, the selection of clients with the possibility of referring them to a psychiatrist may be done properly only if the philosophical counselor has clinical training or has acquired diagnostic skills. Marinoff, also the author of “Philosophical Practice”, acknowledges that the philosophical dialogue is mostly “educational in intent and content, and is neither adversarial nor diagnostic” (Marinoff, 2002, p. 81), reflecting the current opinion in mental health that using diagnostic labels is not useful for the client’s recovery. However, in order to be able to help a psychiatry patient find professional help, the Philosophical Counselor would be advised to use few clinical selection guidelines, also developed in the current paper. The psychotherapy profession is rather easily accessible worldwide, since people with all sorts of BA degrees: theology, sociology, medical school, nursing, psychology, are able to get training in psychotherapy, not always being required to hold a MA degree in psychology or psychotherapy. In a philosophical counseling practice, the selection of the counselee’s has the same degree of the difficulty as in a psychotherapy practice. One of the aspects that may differentiate a philosophical counselor from a psychotherapist is that the philosophical counselor did not acquire in the process of training the diagnostic skills that the psychological practitioner has, which may make the process even harder. Apparently this fact would drastically limit their area of intervention to the ‘normal’^i^, non-symptomatic persons who would only like to have few sessions meant to clarify their thinking regarding personal problems, moral dilemmas, moral conflicts or who would also like to achieve an eudaimonic well-being, in the Aristotelian sense. Eudaimonic well-being reflects traits concerned with personal growth, self-acceptance, purpose in life and autonomy (Ryff, 1995).

For a professional, it is much easier to decide if a person asking for psychotherapy or philosophical counseling should actually be referred to a psychiatrist, since the symptoms of a psychiatric disorder are usually florid and, during the interview, a trained eye could see if the person in front of the practitioner is in reality or is constructing his or her own reality. The problem of selection could get even more complicated when a person comes to a philosophical consultation in order to ask for life advice or for help to solve a moral dilemma. If this person has also emotional disturbances or has previously been diagnosed with depression, anxiety or panic attacks, the philosophical counselor should refer this client either to a psychotherapist or to a psychiatrist. The selection of clients is a common issue also encountered in life coaching, since this sort of overlapping is usual in the counseling professions.

Another situation is also stated in the American Philosophical Practitioners Association’s FAQ about philosophical counseling: “many clients of philosophical counseling have sensibly explored psychology as a prelude to philosophy, that means none of the psychological theories or methods suited them perfectly”; this could lead to the idea that they were dissatisfied with the solution offered by the psychologist or with the psychological approach (American Philosophical Practitioners Association, 2015b). I would argue in this paper that there may also be a lot of clients for whom none of the theories or techniques of psychotherapy work on their particular type of problems (moral or ethical dilemmas) and here is where philosophical counseling or training in ethics may help.

Therefore selection, as simple as it may seem at first glance for an untrained eye, it is actually a difficult process which should be regarded with the highest concern. Emmy Van Deurzen regards the selection issue in a more detached manner, in the sense that: “clients who come specifically for existential therapy usually already have the idea that their problems are about living, and are not a form of pathology” (van Deurzen, 2006, p. 205). In other words, it is mainly the client’s responsibility to assess the type of approach he or she needs. In my view, this responsibility is shared between the counselor (psychotherapist) and the counselee (the client), with an emphasis on the counselor (or the psychotherapist) opinion, considering at least the temporary disorientation or heteronomy of the person entering the therapy or the consultation room.

In the helping professions field there is an increasing need to establish a set of guidelines in order to determine which method should be applied in a specific clinical situation. Moreover, we may consider the opportunity of using an ethical decision making strategy in this process. In this paper I will address the issue of developing a set of criteria when a new client enters the consultation room of a counseling professional, that would help the professional make a correct and informed decision, either keeping the client in his practice or referring him to another practitioner.

In the initial interview or discussion, the client is usually asked first:

1. What is the reason of approaching a particular professional (a psychiatrist, a psychotherapist or a philosophical counselor)?
2. What are the objectives he or she would like to achieve in the sessions?

The interview would be more successful in addressing the client issues if it followed some selection guidelines that are highlighted below.

Guidelines for referring the client to a psychiatrist:

- A psychiatric diagnosis of psychosis on Axis I^ii^ (schizophrenia, bipolar disorder, etc.) and client being currently under medication;
- Even though not been previously diagnosed with a psychiatric condition, the client currently has suicidal ideation or suicidal thoughts. Scores higher than 30 on BDI-II-Beck Depression Inventory indicate a severe depression that should be addressed with the highest care;
- There is evidence of drug addiction or substance abuse, even though the client is undergoing treatment;
- The client suffers from other debilitating symptoms not mentioned above that prevent him or her to function normally and also desires fast symptom relief.

Guidelines for referring the client to a psychotherapist:

- Existence of symptomatology such as hypochondriac complaints, anxiety, phobia, conversive symptoms, somatization, depressive symptoms, that client desires to treat without medication, only via psychological methods;
- There is suspicion of a personality disorder (antisocial, borderline, dependent, etc.) on Axis II^iii^;
- There is a history of child abuse or trauma;
- Even though not previously diagnosed with a psychiatric condition, the scores at BDI-II indicate a mild or moderate depression (lower than 30); severe depression with suicidal ideation is considered a psychiatric emergency;
- Even though the client is currently seen by a psychiatrist for depression or other psychiatric condition, he or she can still receive psychotherapy in order to prevent relapse and learn new coping skills.
- The client would like to have a specialist teach him how to develop skills in order to address issues such as lack of assertiveness, procrastination, self-esteem, how to cope with frustration, with difficult social situations, the type of non-clinical issues that can be addressed with talk therapy.

Guidelines for referring the client to a philosophical counselor:

- The client does not suffer from a psychiatric condition and he is not currently prescribed psychiatric medication;
- In the particular case the patient had a previous psychiatric condition that is in full remission e.g. a depression episode in the past that had been successfully treated using psychotherapy and/or medication, he/she does not have any symptoms at the time being and only wants to investigate other issues that could improve his/her wellbeing;
- The client wants to explore the meaning of his or her life, to explore existential issues, to develop his or her ability to understand personal problems, to solve conflicts or moral dilemmas;
- The client needs to develop critical thinking abilities in practical or theoretical contexts, to identify and eliminate cognitive distortions, argumentation errors and prejudice;
- The client wants to refine his world view, his set of beliefs that guide his daily actions and determine his choices or life options.

The above criteria are drawn largely from the definitions of the three intertwined and frequently overlapping fields of the helping professions. However, perfect and accurate delimitation cannot be made since there will always be an overlapping area between psychiatry and psychotherapy (at least in mild and moderate depression and anxiety) and another overlapping between psychotherapy and philosophical counseling (at least in the existential issues and moral conflicts area), but we can strive for more and more accuracy in the future and hopefully this is the first attempt that will encourage other researchers to study the client selection topic.

### Vignette – Case Study

The client, a 27 years old man working in a corporate environment in a support role, comes into the therapy room with a set of complaints in the existential domain that would make him a good client for therapy, but also for philosophical counseling. His problems are: a not so fulfilling relationship with his partner (who has been for years involved in another relationship that she does not want to quit) and a corporate job that does not fulfill him neither creatively nor intellectually, only financially. There is no indication on Axis II diagnostic and the client had a very happy and protected childhood, no suspicion of infantile trauma whatsoever.

Since the client has not been previously diagnosed with a psychiatric condition and the BDI – II and BAI scores did indicate subclinical depression and anxiety scores, I have not referred the client to a psychiatrist, following his wish that he does not want to use psychiatric medication in order to improve his life. The therapy relied largely on investigating ways in which the client could change his romantic life and also his professional life for the better. The first objective, to improve his personal life, has started with the exploration of reasons for which he still remained in the unfulfilling relationship. Many cognitive distortions and logical errors, especially catastrophizing and black and white thinking have been identified: “I am not good enough to have a woman who loves me for who I am”, “If she cannot give up the relationship with the other man for me, that means I am worthless and I will never find someone to love only me”, “I am not strong enough to remain single until I find someone who only loves me”. Also, exploring the professional life domain, a lot of cognitive distortions have been also identified: “My job is worthless”, “I don’t think that my job has any valuable influence on the society”, “I find hard to secure a job that suits my personality”, “I don’t think that I have any real talents and abilities”, “Staying in front of the PC all day long exhausts me”, “I don’t think my job involves anything remotely creative”. After analyzing all the data the client brought into the counseling room, after formulating his objectives collaboratively and also considering the selection guidelines, in this particular case the client’s problems could also be approached with philosophical counseling methods, therefore both psychological therapy and philosophical counseling would be equally efficient. No need to refer the client to a psychiatrist.

## Moral Dilemmas in Psychotherapy and in Philosophical Counseling

In this section, I will not focus on the key ethical issues that affect the work of a counseling professional, such as autonomy, beneficence, non-maleficence, confidentiality, justice or dual relationships (Vyskocilova & Prasko, 2013), but I mainly on ethical or moral dilemmas that may torment the client’s or the counselee’s lives and usually brings him/her to the consultation room in order to have a resolution.

The client may come in the therapy room or in philosophical consultation room with all sorts of ethical or moral dilemmas that arise from his/her own experience or from his/her experience with others: decisions about whether to stay in a love relationship, how to cope with a divorce and the legal issues linked to it, overcoming lack of satisfaction with one's job, decisions whether to report a corrupt employer (professional ethical conflicts), working through a religious crisis, resolving fights with in-laws, accepting one's sexuality and gender identity, etc.

Ethical dilemmas raised by clients or counselees are the type of dilemmas that can have a huge or a limited impact on their lives, but nevertheless there is not a single psychotherapy client who does not have some sort of moral dilemma unsolved. Pope & Vasquez (2007) mention few typical circumstances encountered in sessions: either the client is in an delicate couple situation following an affair or a divorce, or in a dilemmatic situation in his or her professional life or perhaps thinking of changing’s one religion or gender. There is no guarantee that a philosophical counselor could better solve a moral dilemma than a psychologist with ethical training, but there are reasons to believe that a specialist in ethics is much more equipped at least theoretically to deal with all sorts of situations that may occur in practice that require specialization in ethics.

Many times, in psychotherapy practice, people come with difficult situations that cause them a great deal of anxiety. A common situation is when a client has a friend who is a drug addict and the client does not come easily to terms with the friend’s new status. The client has to face a distinction between “what is legal, what is ethical and what is moral”^iv^, he or she also needs to cope with conflicting values and duties. I believe that this type of dilemma could be solved by a practitioner with ethical training. There is counseling literature suggesting that “it is not useful to avoid dealing with dilemmas by retaining a neutral position and that common-sense-based interventions can be harmful rather than helpful” (van Deurzen, 1999a, p. 581). In order to clarify the client’s conflicts and in the hope of solving their current existential issues, the same author recommends the psychotherapists a more thorough examination of philosophical and moral issues and rely less and less on common sense to guide their interventions.

On the other hand, Lydia Amir’s view on resolving conflict and dilemmas is quite opposed to van Deurzen’s view, suggesting that resolving moral conflicts in the counseling session is not always the case and humor only may help us in a more efficient way to cope with moral conflicts. Amir (2004b) embraces the thesis that humor helps us to get self-knowledge, humor is ambivalent and least, but not last, humor enables deliberation. Compassionate criticism is the key to well-being, not an attitude of obsessively seeking to resolve all conflicts.

Moral dilemmas, both in psychotherapy and philosophical counseling, are occasions to test the practitioner’s skills to help clients and counselees to solve their ambivalence and cognitive dissonance regarding various issues such as inappropriate romantic involvement, jealousy, coping with extra-marital affairs, etc. Sometimes psychologists, blinded by the power of emotionality and less inclined to help clients exercise critical thinking, see those dilemmas more as opportunities to elicit strong emotions in their clients, hoping that the clients themselves will deliberate and get to resolution. They cannot do much in terms of helping them with acquiring new skills to actually resolve the issues that brought them into therapy. Here is where a specialist in ethics or moral philosophy is expected to have a valuable unprejudiced opinion regardless of common beliefs on how things ‘should be done’.

## Existential Issues Encountered in Both Practices

Many influential psychologists or psychiatrists coming from various psychotherapeutic traditions (psychoanalytical, person-centered, rational emotive behavior), such as Jung, Rogers, Ellis, Frankl, Fromm and lately Yalom, moved toward philosophical or existential ways of counseling. Most philosophical counselors and also CBT or REBT therapists have similar views regarding the process:

They appeal to higher aspects of our being than our emotions, such as our reason, which in the long run is a stronger force to be considered in our life. Many clients of philosophical counseling have wisely explored psychology as a prelude to philosophy, that means none of the psychological theories or methods suited them perfectly. (American Philosophical Practitioners Association, 2015a, p. 3).

Currently, Irvin Yalom and Emmy van Deurzen, both famous existential psychotherapists, consider the following philosophical issues or “fundamental worries” as causing many psychological disturbances or life crises: death as a main source of anguish, freedom – responsibility and existential guilt, existential isolation – a new approach of interpersonal relationships and lack of meaning, which may have psychological and philosophical implications (Yalom, 1980). Existential psychotherapists, as opposed to cognitive behavioral therapists, focus also on emotions, both as means of detecting client’s values and as revealing objects of the client’s *Weltanschauung*^v^ (LeBon, 2001). Existential psychotherapy, being the only established form of psychotherapy that is directly based in philosophy rather than in psychology, can take many different shapes and forms, but it always “requires a philosophical exploration of what is true for the client” (van Deurzen, 1999b, p. 232).

### Clinical Observations in Psychotherapy Practice

Following the anamnestic interview and the psychological evaluation session assisted by the use of validated psychological assessment instruments – BDI II (Beck Depression Inventory), BAI (Beck Anxiety Inventory), SCL-90 Symptom Checklist, WHOQOL-BREF 26 items, Inventory of Interpersonal Problems (IIP-64) and Sentence Completion Test (80 items) – I have noticed that behind most of the invalidating symptoms and current life issues or feeling of inadequacy, almost always lies a deeper, most profound cause, that is not necessarily a childhood traumatic event or an adult trauma. Sentence Completion Test (SCT- 80 items) is a psychological instrument of psychodynamic origin and high-face validity. When applying the test to a new client, the following existential issues may arise: meaning of life or lack of it, unclear professional identity, lack of professional identity or identity crisis, fear of death, fear of solitude and loneliness, issues linked to the freedom of choice and liberty. Hypochondriac complaints are mainly linked to fear of death in a less obvious form, as fear of getting a terminal disease, such as cancer. Cancer phobia complaints in their extreme forms are disguised forms of fear of death, which can be dealt with in different manners, using cognitive behavioral techniques or having an existential approach via desensitization to death (Yalom, 1980, p. 16).

There are 14 questions that elicit existential issues in SCT-80:

Q13: The most awful thing that can happen to me is:
Q16: My life:
Q21: I haven’t succeeded in:
Q24: The future:
Q38: When I am honest with myself:
Q43: When I am alone:
Q44: When I work:
Q45: My dreams:
Q57: At my job:
Q65: To be:
Q66: My profession:
Q74: The truth is that:
Q75: I am afraid of:
Q80: To live:

Apart from the obvious phrases or sentences completed that reveal thoughts and considerations on meaning, scope, professional identity, in the actual anamnestic interview the therapist is also expected to explore all the fears experienced by the client and especially the fear of death, the most invalidating of all. The existential psychodynamic emphasizes a different form of fundamental conflict, a conflict “emerged from the individual confrontation with his or her existence” (Yalom, 1980, p. 17).

Together with client’s emotions and cognitions, quality of life was evaluated using WHOQOL-BREF, a 26 items instrument, which measures the following broad domains: physical health, psychological health, social relationships, and environment and also the ORS scale developed by Miller & Duncan, a self-report wellbeing instrument with 4 dimensions: individual, interpersonal, social and global. Depression and anxiety levels were evaluated using the Beck Depression Inventory II and the Beck Anxiety Inventory. Symptom Check-List (SCL-90) is a brief self-report psychometric instrument designated to evaluate a broad range of psychological problems and symptoms of psychopathology, such as somatization, interpersonal sensitivity, depression, anxiety, hostility, etc.

One of my research limitations was that psychological instruments were only used in order to assess the pre-therapy and post-therapy states of the person evaluated and treated. I have not conducted real philosophical counseling sessions, neither have I evaluated the outcome of any philosophical counseling session. Although I am trained in Cognitive Behavior Psychotherapy and Rational Emotive Behavior Therapy, two of the few forms of evidence-based psychotherapies and also two of the therapies that use techniques similar to those used in philosophical counseling, nevertheless I regard the PC approach as a promising avenue for improving psychotherapy.

## Conclusions

Ethical questions and moral dilemmas are an important part of the therapeutic or philosophical counseling process that cannot be neglected. The success of the therapeutic or counseling process depends on ensuring the best practices in the field (either psychotherapy or philosophical counseling). Existential issues are of utmost importance in both types of practices, since issues like meaning, scope, death, freedom and isolation are intrinsic to the human conditions and not psychiatric topics. There is also a multitude of ethical and moral dilemmas to be solved, both in therapy and philosophical counseling, and it is preferable for the counselor or the therapist to have an active role rather than a neutral position. Training recommendations are to be made to the philosophical counselors in order to be able to detect a serious psychiatric condition in a counselee and be able to recommend him or her to a psychiatrist, should this be the case. Also, training recommendations in the ethical field are to be made to the psychotherapist, both in order to improve his or her understanding of ethical or moral dilemmas of the clients or counselees, and also in order to understand ethical challenges of his own field. The client selection issue is a complex one and it is more successfully done if proper training is provided. Future research is needed in order to assess the importance of dilemmas and ethical issues in both practices. Also, future research is needed in order to investigate whether it is possible to achieve in philosophical counseling certain results, such as a superior levels of well-being or quality of life, as in psychotherapy.
